# Association between organizational characteristics of community-oriented mental health facilities and treatment adequacy. A multilevel analysis from Lombardy, Italy

**DOI:** 10.3389/frhs.2025.1655225

**Published:** 2025-11-21

**Authors:** Giovanni Corrao, Matteo Monzio Compagnoni, Claudia Conflitti, Paola Sacchi, Antonio Lora

**Affiliations:** 1 Emeritus Professor of Medical Statistics, University of Milano-Bicocca, Milan, Italy; 2 National Centre for Healthcare Research and Pharmacoepidemiology, University of Milano-Bicocca, Milan, Italy; 3 Welfare Councilor Office, Lombardy Region, Milan, Italy; 4 Unit of Biostatistics, Epidemiology and Public Health, Department of Statistics and Quantitative Methods, University of Milano-Bicocca, Milan, Italy; 5 Unit of Mental Health, Addictions, and Prison Health, Territorial Care Department, Emilia-Romagna Region, Bologna, Emilia-Romagna, Italy; 6 Department of Mental Health and Addiction Services, AUSL Reggio Emilia, Reggio Emilia, Italy; 7 National PhD Programme in One Health Approaches to Infectious Diseases and Life Science Research, Department of Public Health, Experimental and Forensic Medicine, University of Pavia, Pavia, Italy; 8 Territorial Network Organizational Unit; Structure of Mental Health, Addiction Services, Disabilities and Penitentiary Health; Regional Directorate for Health and Welfare, Lombardy Region, Milan, Lombardy, Italy; 9 Department of Mental Health and Addiction Services, ASST Lecco, Lecco, Italy

**Keywords:** mental healthcare, healthcare services, healthcare utilization database, minimally adequate treatment, healthcare research, public health, severe mental illness, multilevel analysis

## Abstract

**Introduction:**

The care provided to patients with severe mental disorders remains a major challenge for the organization of healthcare systems. Data on recent treatment patterns within mental health services are essential to estimate the unmet needs for care and to guide service planning and resource allocation.

**Aim:**

To identify individual patient and organizational-level predictors of the provision of minimally adequate care for patients with severe mental illness.

**Methods:**

A population-based study was designed, retrieving data from Healthcare Utilization databases of Lombardy region (Italy). 72,115 patients from Departments of Mental Health (DMHs) in care for schizophrenic, bipolar or major depressive disorder, were identified. Minimally Adequate Treatment (MAT) was calculated as either minimum psychiatric visits (≥4) with pharmacological treatment (≥2 months) or psychotherapy sessions (≥8, for major depressive disorder only). Patients meeting these criteria were considered as having received MAT; others were classified as having received less than adequate treatment. Multilevel analyses were performed to estimate the association between patients' individual (e.g., age, sex, education, marital status) and DMHs' aggregate (i.e., organizational features, activity volume, staff employed in facilities providing MHC) characteristics and provision of MAT.

**Results:**

Overall, 45% of patients received MAT. Patients with increased probability of receiving MAT included married individuals (8%, 95% CI: 4%–12%), those with schizophrenia (11%, 95% CI: 9%–13%) or bipolar disorder (23%, 20%–25%), younger patients (22%, 20%–25%), and those with previous continuity of care (48%, 46%–51%). Differences in DMHs' structural features (e.g., number of day-treatment facilities, presence of multidisciplinary teams) contributed to heterogeneous MAT coverage. Moreover, the composition of psychiatric teams (in terms of hours worked by each category of healthcare professionals) and the number of affiliated facilities were associated with MAT delivery.

**Conclusions:**

This study ascertained that the quality of care offered to psychiatric patients is still low and not adequate. Administrative data can usefully contribute to identify both individual and organizational-level predictors of MAT provision, offering a valuable benchmark for managing organizational features of DMHs and for optimally allocating the working hours in multidisciplinary professional teams, with the goal of maximizing the provision of adequate mental healthcare.

## Introduction

The World Health Organization includes the provision of mental healthcare in community-based settings as a key objective of Mental Health Care (MHC) systems (1). As a country, Italy offers a unique opportunity to explore community-oriented MHC. Indeed, since the approval of the psychiatric reform law in 1978 (2), a process of deinstitutionalization had led to a profound shift from hospital-based to community-oriented MHC (3). However, community-based MHC requires a continuous effort from healthcare professionals, patients and their families. While adherence to therapies is often suboptimal among people with mental disorders (4–6), inadequate outpatient follow-up further challenges the effectiveness of community-based care. Previous research has identified multiple barriers, including lack of service integration and poor continuity of care, which are associated with worse clinical outcomes, including relapses and re-hospitalizations (7). Indeed, emergency room (ER) visits may indicate suboptimal outpatient management and serve as a marker of inadequate continuity of care, predicting subsequent psychiatric hospitalizations (8, 9). Experience suggests that community-oriented MHC facilities often do not provide adequate care for patients with severe mental illness, resulting in worse prognosis, shorter life expectancy and poor quality of life (10–13). For example, it has been reported that in Italy less than a half of patients affected by severe mental disorders, including schizophrenic, affective and depressive disorders, received adequate care (5, 14–16), and this translates into higher rates of hospitalization and relapses (17). These challenges highlight the importance of developing robust tools to evaluate mental health care.

To assess the quality of MHC, it is essential to define a set of indicators that allow to compare different mental health systems and levels of care within the same system (e.g., primary care vs. specialized services). Among such indicators, the concept of the Minimally Adequate Treatment (MAT) has become relevant (16, 18, 19), and is particularly suitable for the purposes of such comparisons. MAT is typically defined as receiving a sufficient number of psychotherapy sessions or an adequate duration of pharmacological treatment, and it refers to the minimum level of care required to achieve meaningful clinical outcomes (18). A growing body of international evidence suggests that access to MAT is associated with improved clinical outcomes, including reductions in symptom severity, broader impacts on health service utilization and mortality (20). Therefore, ensuring MAT provision to patients with mental disorders is key in evaluating the appropriateness, equity, efficiency, and sustainability of MHC provided by mental health systems.

This paper, moving in the field of retrospective real-world investigation, aims to identify predictors at both the individual patient and organizational levels that influence the provision of the so-called minimally adequate treatment (16, 21–23) for patients with severe mental illness receiving care from the Departments of Mental Health (DMHs) accredited by the Health Service of Lombardy Region. We used multilevel analyses to assess the effect of individual and organizational (POU-level) predictors simultaneously (24). In such analyses, organizational predictors included volume of activity, structural features and staff employed in the facilities providing MHC. From this point of view, the current paper represents an exploratory investigation aimed at supporting policymakers in allocating mental health resources and managing services to optimize the delivery of mental healthcare.

## Methods

### Setting

In Lombardy, an italian region with nearly 10 million inhabitants (around 16% of the whole national population) (25) MHC is currently provided through Departments of Mental Health (DMHs), each of them being structured into Psychiatric Operative Units (POUs). In accordance with the principles established by the Italian psychiatric reform of 1978 (Law 180/1978) (26), which reshaped MHC in Italy, each POU (defined to cover areas with roughly similar populations while accounting for differences in territorial morphology and urbanization) represents a widespread and integrated network of community mental health facilities, including community mental health centres (CMHCs), general hospital psychiatric wards (GHPWs), day-treatment and community residential facilities (27, 28). Each of the 52 POUs currently active in Lombardy at the study time was staffed by a multidisciplinary team that included psychiatrists, psychologists, nurses, social workers, and auxiliary personnel.

### Data source

In Italy, all citizens have equal access to healthcare provided by the National Health Service (NHS). An automated system of healthcare utilization (HCU) databases is used to manage health services in each region. These databases were originally established to record all payments made to healthcare providers for reimbursement purposes, and therefore gathers, on an ongoing basis, administrative and disease-related data for all patients assisted by the Regional Health Services (6). Therefore, HCU data contain a wide range of information on all individuals resident in Lombardy and beneficiaries of the NHS, such as diagnosis at discharge from public or private hospitals, outpatient drug prescriptions, specialist visits and diagnostic exams provided fully, or in part, free-of-charge by the NHS. In addition, a specific national information system dedicated to mental health care is implemented through regional DMH facilities accredited by the NHS. This system gathers detailed information on healthcare services and social interventions provided to patients with mental disorders and their families (5, 29). This information system, called “Mental Health Information System” (MHIS) includes socio-demographic information, diagnostic and therapeutic codes for patients receiving specialist MHC by regional DMH facilities (5). Data on interventions and activities delivered by DMHs in outpatient, home-care, or day-treatment facilities are also recorded within the MHIS.

These various types of data in the HCU databases can be interconnected since a unique individual identification code is used by all databases for each NHS beneficiary, enabling the study of the complete care pathway for each patient. To preserve privacy, each identification code is automatically anonymized, the inverse process being only allowed to the Regional Authority upon request of judicial Authorities. Diagnostic and therapeutic codes, as well as outpatients specialist visits ones, used in the current study for drawing records and fields from Healthcare Utilization databases are reported in the Supplementary Table S1; whereas, the full list of interventions provided by community Mental Health services, as recorded in the MHIS, is reported in Supplementary Table S2. Further details on HCU databases in the field of MHC have been reported elsewhere (12, 14, 29, 30).

In addition to individual-level data obtained through record-linkage procedures across HCU databases, aggregate data on the POUs currently operating in Lombardy were provided by the Regional Directorate for Welfare, with support from their technical IT staff.

### Study population

The target population consisted in all the NHS beneficiaries residents in Lombardy who on 1st January 2015 were aged 18 years or older (about 8.27 million inhabitants in 2015, http://demo.istat.it, last access 22nd May 2025). Of these, individuals who received at least one contact with DMH services (i.e., an hospital admission in a GHPW or a healthcare service at any facility affiliated with DMHs) during the year 2015 were identified, with the date of the first contact with DMH being referred to as the index date. Among those patients, those who received a diagnosis of schizophrenic, bipolar or major depressive disorder were identified and included into the study population. Diagnoses of severe mental disorders were considered as mutually exclusive. For patients with more than one diagnosis, the classification to the most updated one (i.e., the diagnosis recorded in the MHIS at the latest date) was performed by technical staff.

Based on mental healthcare received during the four-year wash-out period prior to the index date (2011–2014), patient histories were examined and patients were classified into three mutually exclusive groups reflecting continuity of care: (i) new patients, who had no previous contact with mental health facilities; (ii) former patients, who had at least one contact with mental health facilities between 2011 and 2013, but none in 2014; and (iii) continuous patients, who received continuous care (one contact per month) throughout the entire period from 2011–2014. Only patients with a minimum of four years of available historical data prior to the index date were included, to avoid selection bias and ensure reliable classification.

Patients included in the study cohort were observed for one year, accumulating person time of follow-up from the index date to the earliest of the following events: death, emigration from the Region or administrative censorship (set at 365 days after the index date; for patients with an index date on 31 December 2015, follow-up could have ended on 31 December 2016).

### Individual and aggregate predictors

Baseline characteristics measured for each patient in the study population (hereafter referred to as “individual-level” data) included sex, age, years of education, employment status and marital status. In addition, physical comorbidities that could act as potential confounders in receiving mental healthcare (e.g., diabetes, hypertension, etc) were assessed both individually and through the Multisource Comorbidity Score (MCS). The MCS is a recently developed comorbidity index that captures patients' clinical status based on inpatient diagnostic information and outpatient drug prescriptions, which has been validated for outcome prediction in several Italian regions (31, 32).

Several characteristics reflecting the organizational features of facilities providing MHC (hereafter referred to as “aggregate” predictors) were assessed for each POU active during the study period, with a brief description of the most relevant structural features of DMHs provided in Supplementary Table S3. Among these predictors, one of the main organizational features of interest was the proportion of hours worked by each professional category related to the total number of hours worked by all professional categories within the POU. For each POU, this proportion was computed as the ratio between the hours worked by each professional category and the total number of hours worked by all healthcare professionals within the same unit. This variable was conceived as an indicator of workforce composition, reflecting the relative weight of each professional category within the multidisciplinary team, as information on the total workforce volume was not available for the analysis. Healthcare professionals were grouped into five categories: (i) medical staff (psychiatrists), (ii) nurses, (iii) psychologists, (iv) social staff (including social workers, educators, and rehabilitation therapists), and (v) auxiliary and administrative staff. Additional aggregate-level predictors of interest included: (i) the number of facilities affiliated with each POU and (ii) the total number of patients under care within the POU.

### Minimally adequate treatment

The so-called Minimally Adequate Treatment (MAT), an indicator developed according to the main recommendations of the American Psychiatric Association (16, 18, 19), was calculated over the one-year observation period for each patient belonging to the study population, and was defined as the outcome of interest.

MAT consists of either a combination of minimum psychiatric and pharmacological treatment, or a minimum number of psychotherapy sessions. For each diagnostic groups considered, a patient was considered as having received MAT if he/she had at least four or more psychiatric visits and was covered for a minimum of two months by appropriate pharmacological therapy (i.e., antidepressants for patients diagnosed with major depressive disorder, antipsychotics for those with schizophrenic disorder, and either antipsychotics, mood stabilizers or both for those with bipolar disorder) (18). Additionally, patients diagnosed with major depressive disorder who attended at least eight psychotherapy sessions were also considered as having received a MAT, even in the absence of psychiatric visits or drug prescriptions (19). Patients not meeting any of these criteria were classified as not having received MAT.

Drug coverage was calculated according with the Defined Daily Dose (DDD) metric. The number of days covered by a given pharmacological class during the 365-day observation period (i.e., the total number of days covered by dispensed drugs belonging to that class) was considered for assessing adherence to drug therapy (33). Hospitalization days and periods spent in residential facilities were considered as days covered by therapy, thereby taking into account the so-called “immeasurable time bias” (34).

### Data analyses

Differences in individual-level characteristics according to MAT provision were investigated. Given the large sample size and the potential for type I error, the standardized mean difference (SMD) was used to identify meaningful differences between patients who did and did not receive MAT. Indeed, SMD is an alternative to the *p*-value and not influenced by sample size (35). Based on previous literature, a threshold of 10% or higher was considered indicative of a relevant difference (35, 36). A multivariable log-binomial model was fitted to estimate the relative risk (RR), and corresponding 95% confidence interval (CI), of MAT provision (outcome) associated with individual-level characters (exposures).

Since one of the main aim was to identify organizational features of POUs associated with MAT provision, and given the clear hierarchical structure of the data, with patients (individual-level) nested within the POUs (aggregate-level), a multilevel log-binomial regression model was fitted. The association between aggregate-level predictors and MAT was assessed by adjusting for individual-level predictors, with POUs included as a random intercept to account for clustering. Between-POUs heterogeneity in the proportion of patients receiving MAT, adjusted for individual-level predictors, was first assessed. As aggregate-level predictors were expressed as continuous variables (see Methods, Individual and Aggregate Predictors section for details), and a non-linear association with outcome was expected, restricted cubic splines with four knots (37, 38) were employed, with the reference value set at the first knot.

All the analyses were performed with the SAS software version 9.4 (SAS Institute, Cary, NC, USA) and the Excel Software (from the Microsoft Office Personal Productivity Software Suite, Version 2019 16.0.6742.2048).

## Results

### Patients

Overall, 72,115 patients were identified, of whom 32,773 (45.4%) received MAT during the one-year observation period. The mean age was 51.6 years (SD = 14.5), and the majority were women (55.6%), employed (54.5%), with no evidence of physical comorbidities (52.9%), and never married (42.7%). Most patients (79.7%) were classified as continuous patients (i.e., those who received ongoing mental healthcare between 2011 and 2014). Regarding primary psychiatric diagnosis, nearly half had a diagnosis of major depressive disorder (47.1%), while 39.4% had a schizophrenic spectrum disorder, and 13.5% had bipolar disorder.

### Individual predictors of minimally adequate treatment

According to the bivariate analysis, younger patients, those who were never married, those with previous continuity of care, and those diagnosed with schizophrenic or bipolar disorder showed, on average, a higher likelihood of receiving MAT compared to their respective comparators (Table 1). No other individual characteristics generated meaningful between-patients differences in our setting. Consistently, according with multivariable log-binomial estimates (Table 2), significantly higher MAT provision was observed among patients with bipolar disorder (+23% than patients with major depressive disorders), patients younger than 29 years (+22% than patients aged 60 years or older), and those with previous continuity of care (+22% compared to newly taken-in-care patients). Other characteristics showed weaker associations with MAT provision compared to the main predictors. For example, male patients had a lower probability of receiving MAT compared to females (relative risk 0.98, 0.96–0.99), patients with disabilities had a slightly higher probability (RR 1.03, 1.00–1.05), while other characteristics showed minimal (RRs close to 1) or non-significant differences.

**Table 1:** Baseline characteristics of patients with severe mental disorder treated by DMHs of lombardy region, according to Minimally Adequate Treatment (MAT) status. Lombardy, Italy, 2015–2016.

	Minimally Adequate Treatment (MAT)		SMD^¥^
	Received MAT	Did not received MAT	
N	32,773 (45.4%)	39,342 (55.6%)	
Age			
Mean (SD)	50.2 (14.1)	52.8 (14.9)	18.1
18–29	2,566 (7.8%)	2,529 (6.4%)	20.6
30–44	8,832 (26.9%)	9,199 (23.4%)	
45–59	12,949 (39.5%)	14,604 (37.1%)	
≥60	8,426 (25.7%)	13,010 (33.1%)	
Sex			
Male	14,773 (45.1%)	17,224 (43.8%)	2.6
Female	18,000 (54.9%)	22,118 (56.2%)	
Continuity of care^§^			
New patients	3,309 (10.1%)	6,001 (15.3%)	29.7
Former patients	1,311 (4.0%)	4,041 (10.3%)	
Continuous patients	28,153 (85.9%)	29,300 (74.5%)	
Clinical profile (MCS)^†^			
Good	17,536 (53.5%)	20,633 (52.4%)	9.9
Intermediate	7,964 (24.3%)	9,142 (23.2%)	
Poor	5,826 (17.8%)	7,550 (19.2%)	
Very poor	1,447 (4.4%)	2,017 (5.1%)	
Type of disorder			
Bipolar disorder	5,158 (15.7%)	4,576 (11.6%)	18.8
Depressive disorder	13,848 (42.3%)	20,126 (51.2%)	
Schizophrenia	13,767 (42.0%)	14,640 (37.2%)	
Education (years)*^γ^*			
0	3,948 (12.0%)	5,670 (14.4%)	7.1
5–8	23,566 (71.9%)	27,275 (69.3%)	
13–18	5,259 (16.0%)	6,397 (16.3%)	
Employment^λ^			
Invalid	6,548 (20.0%)	8,233 (20.9%)	2.6
Unemployed	8,104 (24.7%)	9,931 (25.2%)	
Employed	18,121 (55.3%)	21,178 (53.8%)	
Marital status			
Never married	14,868 (45.4%)	15,893 (40.4%)	13.4
Married	13,167 (40.2%)	16,472 (41.9%)	
Divorced/separated	3,357 (10.2%)	4,612 (11.7%)	
Widowed	1,381 (4.2%)	2,365 (6.0%)	
Level of urbanization^‡^			
Low	3,566 (10.9%)	4,269 (10.9%)	0.0
Medium	15,377 (46.9%)	18,553 (47.2%)	
High	13,830 (42.2%)	16,520 (42.0%)	

SMD, standardized mean difference; MCS, multisource comorbidity score. Data refer to the 2015–2016 period, covering cohort recruitment in 2015 and one-year follow-up.

¥ SMD <0.10 were considered as negligible and to be not statistically significant.

§ Classification in three mutually exclusive groups reflecting continuity of care based on mental healthcare received during the four-year period prior the index date (2011–2014).

† The clinical profile was assessed by using the Multisource Comorbidity Score (MCS), according to the hospital admissions and the drugs prescribed in the two-year period before the index. Four categories of clinical status were considered: good (score = 0), intermediate (1 ≤ score ≤ 10), v (10 < score ≤ 15), and very poor (score > 15).

γ Education (years) was categorized according to the Italian school system: 0 (no elementary school certificate); 5–8 (elementary and lower secondary education); 9–12 (upper secondary education); 13–18 (higher education, i.e., high school diploma, university degree, master's, PhD).

λ Employment status was classified as follows: Invalid; Unemployed (unemployed, looking for a job, student, housewife); Employed.

‡ Level of urbanization retrieved for each local unit from an evaluation of the Italian National Institute of Statistics, ISTAT (year 2018) and classified as low (rural or sparsely populated areas), medium (small cities, suburbs, or intermediate population-density areas), and high (cities or densely populated areas).

**Table 2:** Risk ratio (RR), and corresponding 95% confidence interval (95% CI), for the association between selected characteristics and Minimally Adequate Treatment (MAT) provision. Lombardy, Italy, 2015–2016.

	RR (CI 95%)^¥^	*P*-value^Ω^
Type of disorder		
Depressive disorder	1.00 (Ref)	<.0001
Bipolar disorder	1.23 (1.20–1.25)	
Schizophrenic disorder	1.11 (1.09–1.13)	
Age		
18–29	1.00 (Ref)	<.0001
30–44	0.96 (0.93–0.99)	
45–59	0.91 (0.88–0.94)	
≥60	0.78 (0.75–0.80)	
Sex		
Female	1.00 (Ref)	0.021
Male	0.98 (0.96–0.99)	
Continuity of care^§^		
Continuous patients	1.00 (Ref)	<.0001
Former patients	0.52 (0.49–0.54)	
New patients	0.78 (0.75–0.80)	
Clinical profile (MCS)^†^		
Good	1.00 (Ref)	0.1522
Intermediate	1.04 (1.02–1.06)	
Poor	1.02 (1.00–1.05)	
Very poor	0.98 (0.94–1.02)	
Education (years)^γ^		
0	1.00 (Ref)	0.0777
5–8	1.05 (1.02–1.08)	
13–18	1.04 (1.02–1.08)	
Employment^λ^		
Unemployed	1.00 (Ref)	<.0001
Invalid	1.03 (1.00–1.05)	
Employed	1.04 (1.02–1.06)	
Marital status		
Married	1.00 (Ref)	<.0001
Never married	0.98 (0.96–0.99)	
Divorced/separated	0.93 (0.90–0.95)	
Widowed	0.92 (0.88–0.96)	
Level of urbanization^‡^		
Low	1.00 (Ref)	0.0876
Medium	0.99 (0.97–1.02)	
High	1.01 (0.99–1.04)	

MCS, multisource comorbidity score. Data refer to the 2015–2016 period, covering cohort recruitment in 2015 and one-year follow-up.

¥ Risk ratios (95% CI) estimated by using a log-binomial regression model; the outcome considered was having received the Minimally Adequate Treatment.

Ω P-value for trend (whether the considered categories were ordinal) or for heterogeneity (whether the categories were not ordinal).

§ Classification in three mutually exclusive groups reflecting continuity of care based on mental healthcare received during the four-year period prior the index date (2011–2014).

† The clinical profile was assessed by using the Multisource Comorbidity Score (MCS), according to the hospital admissions and the drugs prescribed in the two-year period before the index. Four categories of clinical status were considered: good (score = 0), intermediate (1 ≤ score ≤ 10), v (10 < score ≤ 15), and very poor (score > 15).

γ Education (years) was categorized according to the Italian school system: 0 (no elementary school certificate); 5–8 (elementary and lower secondary education); 9–12 (upper secondary education); 13–18 (higher education, i.e., high school diploma, university degree, master's, PhD).

λ Employment status was classified as follows: Invalid; Unemployed (unemployed, looking for a job, student, housewife); Employed.

‡ Level of urbanization retrieved for each local unit from an evaluation of the Italian National Institute of Statistics, ISTAT (year 2018) and classified as low (rural or sparsely populated areas), medium (small cities, suburbs, or intermediate population-density areas), and high (cities or densely populated areas).

### Aggregate predictors of minimally adequate treatment

Descriptive statistics for key POU-level characteristics are provided in Supplementary Table S4. Between POUs differences were observed in structural features and activities. Medical staff, nurses, psychologists and social workers contributed, on average, 17%, 44%, 5%, and 16% respectively of the total employed hours, with ranges from 8%–28%, 3%–67%, 0%–11%, and 3%–60%, respectively. Additionally, the number of patients taken in case by the POUs ranged from 493–5,923, with a mean of 2,103, while the number of affiliated facilities per POU ranged from 1–25, averaging 10.

Estimates of the adjusted probability (and 95% CI) of receiving MAT according to the POI where patients were taken into care are shown in Supplementary Figure S1. Each horizontal bar represents a specific unit, highlighting a great variability among POUs, with values ranging from a 60% reduction (95% CI, 48%–68%) to a 20% increase (9%–30%) compared with the regional average. Notably, among the 52 considered POUs, 17 (32.7%) showed probability of providing MAT significantly different with respect to the regional average, with ten of them exhibiting a significantly lower probability of delivering MAT to their patients.

**Figure 1:**
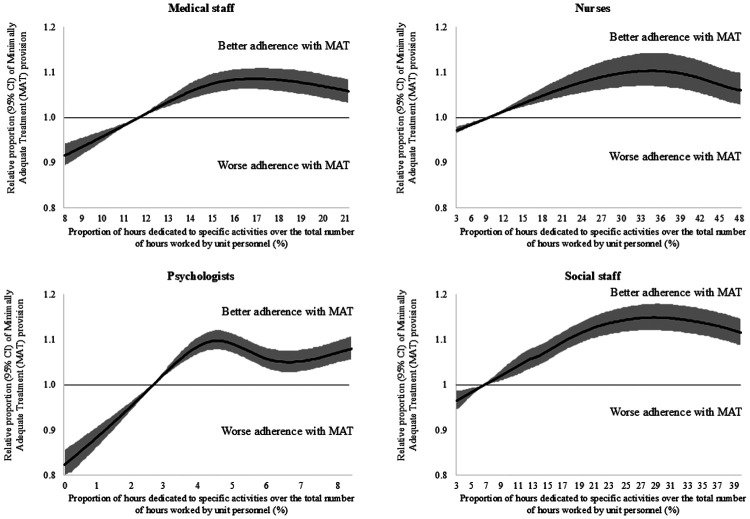
Restricted cubic splines to flexibly modelling the relationship between the proportion of hours worked by each professional category (on the total number of hours worked by all professional within the POU) and relative proportion of patients receiving minimally adequate treatment.

**Figure 2:**
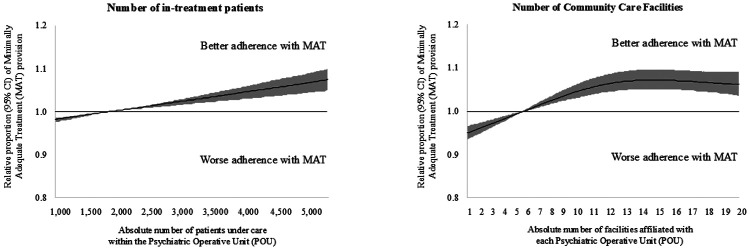
Restricted cubic splines to flexibly modelling the relationship between selected psychiatric operating units structural features (i.e., the number of patients in care, left panel, and of affiliated facilities, right panel) and relative proportion of patients receiving Minimally Adequate Treatment.

As shown in Figures 1, 2, a portion of the observed between-POU heterogeneity can be explained by the considered aggregate predictors which reflect POUs organizational and structural differences. Figure 1 illustrates the patterns of MAT provision according to the proportion of hours employed by each category of healthcare professionals. When the percentage of hours worked by each category was low, the relative proportion of patients receiving MAT remained below one (indicating, on average, the provision of inadequate treatment). Then, as the proportion of hours worked by healthcare professionals increased, this probability of MAT provision progressively increased before stabilizing. Considering the distinct categories of healthcare professionals, the highest relative proportions of patients receiving MAT were reached when: 16% of the total hours were provided by psychiatrists (with an increase of +8.5%; 95% CI: +6.3% to +10.9%), 34% of the total hours were provided by nurses (+10.4%; +6.4% to +14.2%), 4% by psychologists (+9.8%; +7.7% to +12.6%), and 29% by social workers (+14.8%; +12.1% to +17.8%). Figure 2, left panel, shows that higher numbers of patients treated at a POU (i.e., greater POU's activity volumes) were associated with larger increases in MAT provision, with the highest value (+9.5%; +6.1% to +12.8%) being reached by structures caring at least 5,900 patients. Finally, Figure 2, right panel, shows the relationship between the number of affiliated facilities per POU and treatment adequacy. POUs composed by less than 5 affiliated facilities provided inadequate treatment (i.e., relative proportions of MAT provision under the unit), while the relative proportions of patients receiving MAT progressively increased up to POUs with 13 affiliated facilities (gain of +7.2%; 95% CI +5.0% to +9.4%).

## Discussion

The results of the present study offer evidence on the association between several individual-level characteristics of patients with severe mental disorders and the adequacy of mental healthcare provided. Consistently, we observed that new patients were more likely to meet the criteria for minimally adequate treatment (MAT), while patients with longer histories of care may often receive only medication monitoring. In particular, patients who were newly taken-in-care, those diagnosed with major depressive disorders, older patients, and those with a history of treatment interruption in the last few years had a lower probability of receiving minimally adequate treatment (MAT). As a novel and original message, our approach aimed to explore the role of organizational features of mental healthcare facilities. It showed that psychiatric units with low activity volumes and those with fewer territorial structures belonging to them, were less likely to provide minimally adequate treatment to patients they assist. Moreover, our findings may provide a preliminary reference for the distribution of working hours among multidisciplinary professionals to maximize the provision of adequate mental health care. Taken together, these results must be considered a valuable resource for policymakers for appropriately allocating resources to maximize benefits for patients.

Several additional findings of our study deserve to be mentioned. First, accordingly with previous investigations (16, 18, 28, 39), we confirmed that adequacy of community-oriented MHC is not largely achieved, with less than half of patients treated in public facilities in Lombardy receiving minimally adequate treatment. Second, individual-level factors influencing MHC adequacy were only partially consistent with literature: older age and major depressive disorders are well-known risk factors for receiving inadequate treatment and experiencing worse outcomes (16, 40). Conversely, unlike other studies (41–45) we did not find a significant association between social and educational characteristics and the adequacy of care provided. Finally, previous investigations found no clear impact of selected organizational features of Mental Health Departments of Lombardy (e.g., number of psychiatric beds and employed hours in community facilities) on the adequacy of care provided (16). However, it should be considered that in the current investigation was made a great effort to improve the accuracy of metrics measuring organizational features. For example, given that healthcare professionals may work across multiple facilities, we characterized each psychiatric unit based on the percentage of hours worked (an information not easy to retrieve), rather than simply the number of staff, offering a more precise metric.

### Strengths and limitations

The study presented several strengths. HCU databases carry high-quality individual information on outpatient and inpatient services provided by the NHS (46). These data, linked to those collecting information for care provided by public DMHs (e.g., the MHIS), offer the opportunity to trace and evaluate the complete care pathway of patients with severe mental disorders. Thus, administrative databases are a useful tool to reflect routine clinical practice and to generate reliable real-world evidence on mental healthcare (14). Moreover, studies based on HCU data are not affected by selective participation or recall bias. Furthermore, since in Italy a free healthcare system is available to all citizens, using HCU data allow to perform studies on large, unselected cohorts. As a consequence of that, the present population-based study offers guarantees of representativeness and generalizability.

However, the use of administrative databases also presents several limitations. First, HCU data do not capture services provided in primary care settings, private facilities or those paid out-of-pocket (47–49). Moreover, using HCU databases the drug use is based on the assumption that the prescription (and its purchase) corresponds to the real consumption, and this may lead to a possible exposure misclassification. Despite this, HCU data were originally established to reimburse healthcare providers, and an incomplete or incorrect reporting leads to legal consequences, therefore assuring a high quality for the data source (50). Second, the lack of socio-economic and clinical data, such as the severity of the disease, physical complications and comorbidities, represents another important limitation. Indeed, the inability to account for socio-economic and physical characteristics (such as deprivation, BMI, blood pressure, etc.), and procedures not traced by the HCU data (e.g., private outpatients visits) represents an important boundary that doesn't allow to fully explore the complexity of mental disorders and their physical, mental and socio-economic implications. Furthermore, the absence of data on patients with drug addiction limited analyses for this vulnerable subgroup (51). Nevertheless, although patients with multiple mental disorder diagnoses could not be explicitly identified, the Multisource Comorbidity Score (MCS) was used to account for clinical complexity, integrating information from previous diagnoses and prescriptions over the two years prior to the index date. Additionally, the classification into continuity-of-care groups relied on a fixed four-year retrospective window, and only patients with at least four years of available historical data prior to the index date were included, to ensure reliable classification and minimizing selection bias.

Despite the robust study design and the use of advanced statistical methods, as an observational study, the possibility of residual confounding cannot be completely ruled out. Factors such as lifestyle behaviors (e.g., smoking, physical activity, alcohol consumption) or other contextual influences (e.g., work schedules, family responsibilities, or transportation barriers) may still affect treatment delivery). Fourth, while the concept of minimally adequate treatment has been developed in the setting of community surveys, its measurement using secondary administrative data poses challenges for several reasons: (i) individual care needs vary based on clinical, demographic, and social factors, many of which are not captured in HCU databases; (ii) the adequacy of care is assessed only through psychiatric/psychotherapist visits and pharmacological therapy, thus not considering the whole complexity of severe mental disorders and the need for continuous care they often require; (iii) psychosocial interventions are in this way excluded, even though their effectiveness have been largely demonstrated (19). Finally, our approach allowed for the assessment of the effect of the number of hours worked by each professional category only within the limits of the observed data. For example, a limited number of psychologists was employed in each POU of Lombardy, resulting in a low variability of hours compared to other professional categories; due to the absence of a comparator with a higher proportion of hours worked by psychologists, it is not possible to make inferences or logical extrapolations about the effect of a greater contribution beyond the observed range. In light of these considerations, further high-quality investigations are needed to confirm these findings.

## Conclusions

Patients with severe mental illness are, and should remain, one of the primary focusses of the mental health community-based system. However, the low quality of care that many psychiatric patients still receive underscores the urgent need for action at both local and regional levels. To improve community care and ensure adequate support for those who need it most, a data-driven approach must be embraced. “*Let us be driven by data for reducing uncertainty*” should become the guiding principle for supporting policymakers to improve community mental health services.

## Data Availability

The datasets presented in this article are not readily available. Indeed, the data that support the findings of this study are available from the Region of Lombardy, but restrictions apply to the availability of these data, which were used under license for the current study, and so are not publicly available. Data are however available from the authors upon reasonable request and with permission of Lombardy Region. Requests to access the datasets should be directed to giovanni.corrao@unimib.it.
