# Supplementary material for: Association between organizational characteristics of community-oriented mental health facilities and treatment adequacy. A multilevel analysis from Lombardy, Italy
Source: Front Health Serv. 2025 Nov 21;5:1655225. doi: 10.3389/frhs.2025.1655225 (PMC12678280; doi:10.3389/frhs.2025.1655225)

Supplementary Table S1. Diagnostic (ICD-10) codes used in the current study for drawing records and fields from Healthcare Utilization databases.

MAJOR DEPRESSIVE DISORDER

	**ICD-10 codes**
Depressive episode	F32.*
Recurrent depressive disorder	F33.*
Dysthymia	F34.1
Other persistent mood [affective] disorders	F34.8
Persistent mood [affective] disorder, unspecified	F34.9
Other recurrent mood [affective] disorders	F38.1
Other specified mood [affective] disorders	F38.8
Unspecified mood [affective] disorder	F39.*
Post-traumatic stress disorder	F43.1
Adjustment disorders	F43.2

SCHIZOPHRENIA SPECTRUM DISORDER

	**ICD-10 codes**
Schizophrenia	F20.*
Schizotypal disorder	F21.*
Delusional disorders	F22.*
Brief psychotic disorder	F23.*
Shared psychotic disorder	F24.*
Schizoaffective disorders	F25.*
Other psychotic disorder not due to a substance or known physiological condition	F28.*
Unspecified psychosis not due to a substance or known physiological condition	F29.*

BIPOLAR DISORDER

	**ICD-10 codes**
Manic episode	F30.*
Bipolar affective disorder	F31.*
Cyclothymia	F34.0
Other single mood [affective] disorders	F38.0

Supplementary Table S2. Codes used in the current study for drawing records and fields from Healthcare Utilization databases regarding drug therapies (ATC) and service interventions, treatments and activities delivered by Community Mental Health Centers and Day-Treatment facilities (according to their classification in the Italian Mental Health Information System).

DRUG THERAPIES

	**ATC codes**
Antidepressants	N06A
Antipsychotics	N05A (excluded N05AN)
Mood stabilizers	N05AN, N03AX09, N03AF01, N03AG01

OUTPATIENT VISITS
(delivered by territorial and day-treatment facilities of CMHCs)

	**Italian Mental Health Information system codes**
Psychiatric visit	01
Psychotherapy	
Psychological visit	02
Individual psychotherapy	07
Couple psychotherapy	08
Family psychotherapy	09
Group psychotherapy	10
	**Regional outpatient procedure codes**
Psychiatric visit	94.12.1, 94.19.1
Psychotherapy session	94.09, 94.3, 94.42, 94.44

Supplementary Table S3. Aggregate-level organizational structural features of Psychiatric Outpatient Units (POUs) included in the analysis.

Variable name	Description	Data source	Aggregation level
Number of affiliated facilities	Total number of outpatient and day-care facilities formally affiliated with each Psychiatric Operational Unit (POU).	Regional HCU databases	POU
Total patients under care	Number of single patients receiving care in each POU during the study period.	Regional HCU databases	POU
Catchment area population size	Number of inhabitants served by each POU, based on the territorial jurisdiction assigned by the Regional Health Authority.	Regional Health Authority	POU
Workforce composition	Proportion of hours worked by each professional category (psychiatrists, nurses, psychologists, social staff, administrative staff) relative to the total hours worked by all professionals within the POU.	DMH general management offices	POU
Total number of beds	Number of beds for each facility affiliated with each POU, according to the different types: GHPW semi-residential, GHPW day-hospital facilities.	Regional Health Authority	POU
Presence of intensive treatment units (MAUs)	Binary indicator (Yes/No) reflecting the presence of one or more Moduli ad Alta Intensità di Cura (high-intensity treatment units) within the DMH.	DMH general management offices	DMH

DMH: Department of Mental Health. HCU: HealthCare Utilization. POU: Psychiatric Operative Unit. MAU: Moduli ad Alta Intensità di Cura. GHPW: General Hospital Psychiatric Ward.

Footnote. This table reports the main organizational and structural characteristics of the DMHs participating in the study. Variables were collected from the regional health information system (ARIA S.p.A.) and, when not available, obtained directly from the DMH general management offices. Each variable is described in terms of definition, data source, and level of aggregation. These features were included in the analysis as aggregate-level predictors to account for contextual differences across Psychiatric Operational Units (POUs), including structural and workforce composition indicators (e.g., distribution of professional categories, number of affiliated facilities, total patients under care, and presence of intensive treatment units).

Supplementary Table S4. Descriptive statistics of aggregate-level characteristics of Psychiatric Outpatient Units (POUs).

**Aggregate-level characteristic**	**Mean (SD)**	**Min**	**Max**
Numbers of facilities ¥	10.3 (5.4)	1	25
Number of MAUs Ω	23.5 (20.4)	6	70
Number of patients taken-in-care ¥	2,103 (937)	493	5,923
Total population ¥	176’394.1 (77’322.0)	93’071	510’400
Total number of beds ¥	42.7 (27.1)	14	188
Beds in GHPW	13.3 (6.2)	0	36
Beds in GHPW - DH	1.0 (2.4)	0	25
Beds in residential facilities^	26.4 (17.1)	0	86
Proportion of hours worked by each professional category ¥			
Medical staff	16.7% (4.2)	8.4%	27.6%
Nurses	44.1% (13.9)	2.5%	65.8%
Psychologists	4.8% (2.2)	0.0%	11.1%
Psychosocial staff	16.0% (11.4)	3.2%	59.6%

POU: Psychiatric Operative Unit. SD: Standard Deviation. MAU: Moduli ad Alta Intensità di Cura. GHPW: General Hospital Psychiatric Ward. DH: Day-Hospital.
¥ Level of Aggregation: Psychiatric Operative Unit (POU).
Ω Level of Aggregation: Department of Mental Health (DMH).

Supplementary Figure S1. Random effect estimates of the ratio between the proportion of patients receiving Minimally Adequate Treatment in each psychiatric operating unit (POU) and the average observed in the entire region.

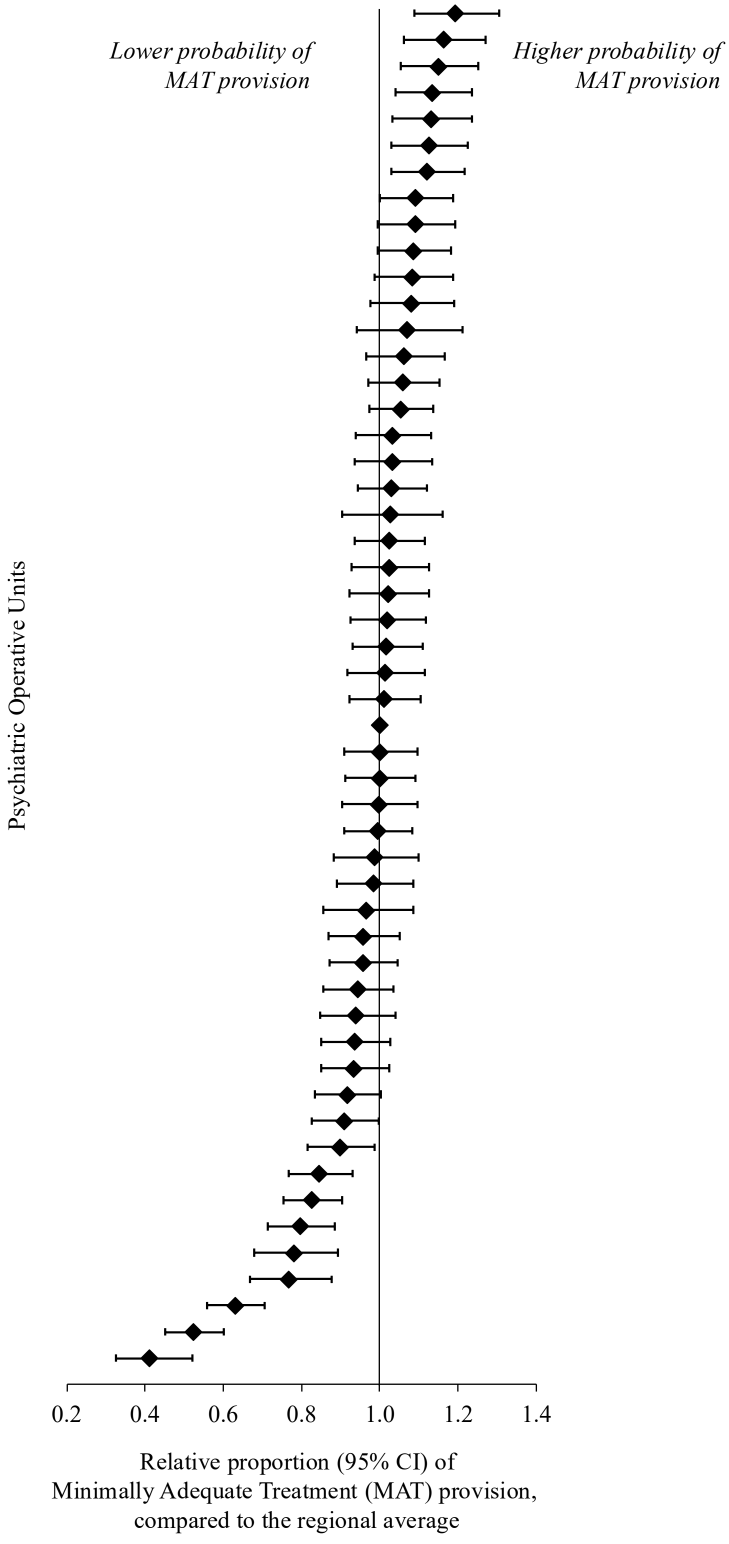

Supplement: Supplementary file 1 [file Datasheet1.pdf]
